# Sex, hormones and cerebrovascular function: from development to disorder

**DOI:** 10.1186/s12987-023-00496-3

**Published:** 2024-01-04

**Authors:** Adeline Collignon, Laurence Dion-Albert, Caroline Ménard, Vanessa Coelho-Santos

**Affiliations:** 1 Department of Psychiatry & Neuroscience and CERVO Brain Research Center, Universite Laval, Quebec City, Canada; 2 Institute for Nuclear Sciences Applied to Health (ICNAS), University of Coimbra, Coimbra, Portugal; 3 University of Coimbra, Coimbra Institute for Biomedical Imaging and Translational Research (CIBIT), Coimbra, Portugal; 4 Faculty of Medicine, University of Coimbra, Institute of Physiology, Coimbra, Portugal

**Keywords:** Autism spectrum disorder, Attention-deficit/hyperactivity disorder, Neurovascular development, Neurovascular unit, Neurodevelopmental disorders, Gonadal hormones, Sex differences, Cerebral blood flow

## Abstract

Proper cerebrovascular development and neurogliovascular unit assembly are essential for brain growth and function throughout life, ensuring the continuous supply of nutrients and oxygen. This involves crucial events during pre- and postnatal stages through key pathways, including vascular endothelial growth factor (VEGF) and Wnt signaling. These pathways are pivotal for brain vascular growth, expansion, and blood–brain barrier (BBB) maturation. Interestingly, during fetal and neonatal life, cerebrovascular formation coincides with the early peak activity of the hypothalamic-pituitary-gonadal axis, supporting the idea of sex hormonal influence on cerebrovascular development and barriergenesis.

Sex hormonal dysregulation in early development has been implicated in neurodevelopmental disorders with highly sexually dimorphic features, such as autism spectrum disorder (ASD) and attention-deficit/hyperactivity disorder (ADHD). Both disorders show higher prevalence in men, with varying symptoms between sexes, with boys exhibiting more externalizing behaviors, such as aggressivity or hyperactivity, and girls displaying higher internalizing behaviors, including anxiety, depression, or attention disorders. Indeed, ASD and ADHD are linked to high prenatal testosterone exposure and reduced aromatase expression, potentially explaining sex differences in prevalence and symptomatology. In line with this, high estrogen levels seem to attenuate ADHD symptoms. At the cerebrovascular level, sex- and region-specific variations of cerebral blood flow perfusion have been reported in both conditions, indicating an impact of gonadal hormones on the brain vascular system, disrupting its ability to respond to neuronal demands.

This review aims to provide an overview of the existing knowledge concerning the impact of sex hormones on cerebrovascular formation and maturation, as well as the onset of neurodevelopmental disorders. Here, we explore the concept of gonadal hormone interactions with brain vascular and BBB development to function, with a particular focus on the modulation of VEGF and Wnt signaling. We outline how these pathways may be involved in the underpinnings of ASD and ADHD. Outstanding questions and potential avenues for future research are highlighted, as uncovering sex-specific physiological and pathological aspects of brain vascular development might lead to innovative therapeutic approaches in the context of ASD, ADHD and beyond.

## Introduction

Brain functions require adequate and constant blood supply of nutrients and oxygen, which is provided by a complex vascular network [1]. These continuous and non-fenestrated vessels ensure the homeostatic balance and a certain immune privilege to the brain [2]. This is not a one-man job, but rather a team effort among vascular cells, such as brain endothelial cells (BECs), mural cells including pericytes (PCs) and vascular smooth muscle cells (VSMCs), and astrocytes. Together, they form a tightly regulated and dynamic interface known as the blood–brain barrier (BBB) [3–5].

BECs are the first frontier between the periphery and the brain. Tight junction proteins, like claudins and occludins, seal the gaps between BECs, effectively blocking potentially harmful peripheral substances such as toxins, pathogens, and immune cells from entering the central nervous system (CNS) ([6, 7], see [8] for review). The brain endothelium is also equipped with specialized transport systems, enabling precise regulation of nutrient and water exchange to maintain brain homeostasis ([7, 9, 10], see [2] for review). Vessel ensheathment by PCs further promotes differentiation and maturation of BECs. In addition, PCs enhance BBB integrity [11, 12], by initiating astrocytic polarization and attachment of their endfeet to the endothelium ([13], see [8] for review). Astrocyte foot processes establish contacts with BECs and mural cells where they contribute to BBB integrity by modulating tight junction expression and regulating water transport to the brain through aquaporin-4 channels [14–19].

The BBB is supported by the neurogliovascular unit (NVU), which also includes microglia and neurons in addition to the specialized BEC, PCs and astrocytic endfeet. The basement membrane additionally regulates structural integrity and intercellular crosstalk between the cells of the NVU, as it covers both BECs and PCs and is surrounded by astrocytes endfeet [20]. The synchronized activity of NVU cells ensures efficient regulation of cerebral blood flow (CBF) to respond to metabolic demands in response to neuronal activation, in a process called neurovascular coupling [21–23]. It also allows waste clearance and neuroimmune responses [24, 25].

### Cerebrovascular development and barriergenesis

**Fig. 1:**
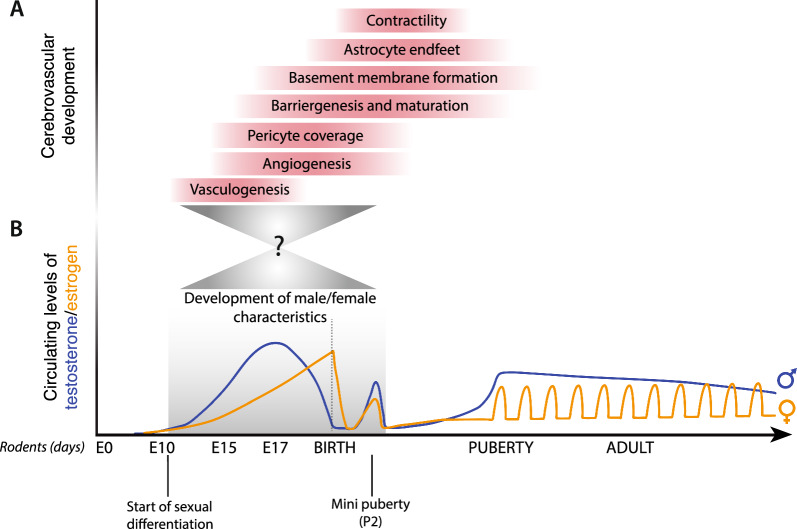
Cerebrovascular development and corresponding circulating levels of sex hormones throughout life in mice **A** Schematic timeline illustrating cerebrovascular development and neurogliovascular unit cells assembly events. Cerebrovascular development in mice begins during the embryonic stage ~ E9-E10. Brain endothelial cells (BECs) start to invade the neuroectoderm in response to local gradients of factors like vascular endothelial growth factor (VEGF). This invasion initiates the formation of primitive blood vessels within the developing brain. Between E11-E14, angiogenesis involves the sprouting of new blood vessels from pre-existing ones, contributing to the expansion of the brain vascular network. Blood–brain barrier (BBB) formation occurs between E12 and E17. During this period, tight junctions seal BECs, restricting the movement of substances across the endothelial cell layer. Between E13 and E17, pericytes wrap around BECs, contributing to the cerebrovasculature structural stability, while a basement membrane arises around blood vessels, providing additional support. After birth, brain capillary networks expand and mature, with astrocyte endfeet helping to maintain BBB integrity and actively supporting the cerebrovascular system throughout life. **B** Schematic illustration (not to scale) of sexual differentiation and circulating sex hormone levels from embryogenesis until adulthood. Man and woman characteristics start to develop ~ E10 (gray rectangle), via progressive elevations of testosterone in male mice (blue) and estrogen in female mice (orange) embryos. After birth, levels quickly decrease, to rise again around postnatal day 2 (P2), a period referred to as mini-puberty. In both sexes, gonadal hormone levels slowly escalate until puberty, which is characterized by monthly estrogenic cycling in women until menopause while in men, testosterone peaks then slowly decline yearly throughout adulthood. Testosterone cycling in women and estrogen cycling in men were not represented for simplicity. Early sexual differentiation coincides with several steps of cerebrovascular development, raising questions regarding potential interactions underlying sexually dimorphic cerebrovascular development (gray triangles)

Proper NVU assembly and vascular integrity are necessary to sustain neuronal activity and behavioral regulation across lifespan. Thus, appropriate formation of this barrier during pre- and postnatal development is critical (Fig. 1A). During early embryogenesis, de novo formation of vessels from endothelial progenitor cells (also known as angioblasts) sets the primary vascular plexus. This process is called vasculogenesis and occurs around embryonic (E) day 9.5 in the mouse embryo, which corresponds to 4–7 gestational weeks in humans [26–28]. Subsequently, by E11-E14 in mice, correlating to about 7 gestational weeks in humans, angiogenic sprouting from the pre-existing vessels forms the perineural vascular plexus (PNVP) and invade the neural tube [29–31]. Sprouting BECs respond to local gradients of secreted factors by neural progenitor cells, such as vascular endothelial growth factor (VEGF) and Sonic hedgehog (Shh) [32, 33], see [8] for review). This process is critical for expanding the vascular network within the developing brain, supporting the increasing metabolic demands of growing neural tissue. Mechanical forces induced by early blood flow contribute to creating vascular patterns [34]. Barrier formation is further carried out by the Wnt/β-catenin signaling pathway, which supports formation of tight junctions, elimination of fenestrations, and restriction of pinocytosis [35]. To support the growing metabolic demands in nutrients, transporters are functional as soon as the BBB emerges in early stages of development [36]. In mice, this transport system has appeared by E13.5 allowing clearance of waste and toxic substances from the brain without preventing the entrance of nutrients and metabolites ([36], see [8] for review). The interval encompassing E13-E18 witnesses the association of pericytes with developing blood vessels. These cells play a pivotal role in conferring structural stability to the emerging cerebrovasculature [37]. Then, additional cell types are recruited to initiate barrier maturation into the adult BBB (Fig. 1A). After birth, cerebrovascular development continues through the postnatal period. In mice, this extends from postnatal day 0 (P0) to P25, while in humans, it spans from birth into infancy [38, 39]. During this phase, the existing vasculature further matures [40, 41]. For example, astrocytes continue to contribute to the structural and functional aspects of the BBB [42, 43], by upregulating tight junction protein expression through Shh signaling in BECs [32], a crucial step to stabilize the vasculature and promote BBB integrity (see [44] for review).

Effective bidirectional communication between the neuronal and vascular system during brain development is essential for proper maturation [45–48]. Unlike the adult CNS, the neonatal brain displays absent or even inverted hemodynamic responses to neuronal activity [49]. While neuronal activity generally consumes oxygen, local vascular responses are insufficient to supply additional oxygen in the developing brain. Hemodynamic responses gradually adapt postnatally [49], coincidently with expansion of the capillary network [40], development of VSMCs contractility capacities [41] and a steady increase of CBF [40, 50, 51]. To this day, the exact mechanisms underlying establishment of hemodynamic responses in the neonatal brain remain poorly understood.

### Neurodevelopmental disorders: how does sex influence cerebrovascular development?

Brain vascular dysfunctions are increasingly explored in the onset and progression of neurodevelopmental disorders, with mounting evidence linking BBB disruption to conditions such as schizophrenia [52], autism spectrum disorder (ASD) [53] and attention deficit hyperactivity disorder (ADHD) ([54], see [55–57] for review). While it is generally accepted that women are protected from vascular dysfunction (e.g. stroke, cardiovascular diseases, etc.) throughout reproductive years due to sex hormones, little is known regarding the mechanisms underlying estrogen-mediated vascular protection, and very little emphasis has been put to decipher the effects of sex hormones on cerebrovascular development. Considering that several neurodevelopmental conditions, including ASD and ADHD predominantly affect men, it is reasonable to suggest that specific mechanisms come into play during cerebrovascular development to sustain this sex bias.

In this review, we first describe processes occurring during early sexual differentiation and how they could modulate important stages of cerebrovascular development. We specifically highlight two major signaling pathways involved in angiogenesis and BBB development, namely the VEGF and Wnt/β-Catenin pathways, with a specific focus on their interaction with gonadal hormones. We next discuss the role of these relationships in vascular dysfunction observed in two major neurodevelopmental conditions, ASD and ADHD. We chose to focus on findings related to BECs, however steroid hormones can also act directly on other NVU components such as mural and glial cells, suggesting an indirect impact on cerebrovascular function ([58–60], see [61–63] for review). Due to limited amounts of literature available, we could only speculate about associations and propose hypotheses regarding hormone-mediated effects on cerebrovascular development, both in the context of health and disease. We shed light on pitfalls of current research, as we firmly believe that advancing knowledge in these areas is crucial for the development of novel screening tools, personalized standardized diagnostic tests, and next-generation pharmacological therapies.

## Sex hormones and cerebrovascular function

### Gonadal development and early sexual differentiation

The hypothalamic-pituitary-gonadal (HPG) axis regulates gonadal hormone production and secretion. In women, the predominant hormones are progesterone and estrogens. Progesterone interacts with the progesterone receptor (PGR) [64], while estrogen acts on BECs through three main receptors: estrogen receptor alpha (Erα), beta (Erβ) and 7-transmembrane spanning G-protein coupled receptor 1 (GPER1, also known as GPR30) [65–67]. In men, androgens like testosterone bind to the androgen receptor (AR) and represent the main hormonal effectors. Gonadal hormones are small, lipophilic molecules that can either be endogenously produced in the brain or diffuse through the BBB [68], (see [69] for review). Once in the brain, binding of gonadal hormones to their receptors activate downstream pathways regulating genomic and non-genomic actions [70, 71].

During the fetal period, HPG axis is most active around mid-gestation, before being silenced prior to birth leading to a decline of gonadal hormone levels (Fig. 1B). At birth, the axis is reactivated, leading to the release of gonadotropins by the pituitary gland to allow sex hormone secretion from gonads, in both sexes [72–75]. This early postnatal period, referred to as mini puberty, occurs concomitantly with rapid brain development. These hormonal fluctuations could participate in sex differentiation of the brain, and shape underlying morphology and behavior, (see [76, 77] for review). Indeed, significant differences in brain volume and region-specific dimorphisms between sexes have been reported in adults, particularly in the cortex [78]. Clustering of sexual dimorphism in the adult brain is observed mainly in areas involved in early sexual differentiation and known to express high levels of gonadal hormone receptors during critical periods of development [78]. Thus, factors implicated in in utero and early postnatal sexual differentiation may underlie region-specific differences in the adult brain of men and women [78] emphasizing that sex as a biological factor should be carefully considered when studying brain development, function, and behavioral regulation [79].

Although beyond the scope of this review, it is important to acknowledge that sex hormones might not be uniquely responsible for brain differentiation according to sex. For example, Dewing et al. [80] performed microarray analysis of male and female mouse brain tissue at 10.5 days post-coitum, a stage prior to gonadal formation. They identified several genes differentially expressed, suggesting that sex determining genetic factors (i.e. XX or XY chromosomes) may underlie brain sexual differentiation prior to gonadal influence [80].

### The brain endothelium as a target for sex hormones: what about the developing brain?

Brain blood vessels are known to be a target for sex hormones, as they express Erα, Erβ, GPeR1 and ARs ([59, 81, 82], see [83] for review). Numerous in vitro and adult rodent studies have confirmed the capacity of estrogens and androgens to modulate angiogenesis, BBB permeability and vascular tone (see [83, 84] for review). These effects are highly age- and dose-dependent, as outlined in Table 1. Estrogen, in particular, is widely recognized for its protective effects on the vasculature, especially while estrogen levels are high during the reproductive years ([85, 86], see [87, 88] for review). This may contribute to sex differences in the prevalence of several disorders. Neuroanatomical studies performed in rodents revealed differential distribution of brain ARs and ERs from early postnatal to prepubertal stages [89–91]; however, the functional role of these receptors and their specific expression in brain vasculature during brain development have been poorly defined and relevance to humans is undetermined.

**Table 1:** Evidence of protective vascular effects of sex hormones in vitro and in adult rodent studies

Hormone	Sex & model	Age	Finding	Refs
Estrogen	in vitro Human vascular BECs cultures exposed to 17β-estradiol	N/A	17β-estradiol induced biphasic effect on tight junctions and paracellular permeability	[228]
	in vitro Mouse brain microvascular endothelial cell cultures exposed to 17β-estradiol	N/A	Identification of an estrogen-responsive element at the Claudin-5 protein promoter, confirming the ability of 17β-estradiol to modulate BBB permeability	[84, 228]
	in vivo Ovariectomized (OVX) female mice treated with estradiol via subcutaneous osmotic mini-pump ERβ knockout female mice	3-months old	Estradiol-treated ovariectomized mice had significantly increased levels of Claudin-5 protein in the brain, compared to sham-operated mice Brain levels of Claudin-5 were significantly decreased in ERβ knockout vs. wild type animals	[228]
	in vivo Female rats after ovariectomy (OVX) + estrogen replacement	Young (4 months) vs old (9–11 months)	Estrogen replacement rescued BBB integrity in young but not old female rats	[229]
Testosterone	in vivo Gonadectomized male mice treated with testosterone via subcutaneous implants (5 weeks)	2-months old	Gonadectomy increased BBB permeability and altered expression of tight junction proteins in the medial preoptic area. Testosterone supplementation rescued BBB integrity	[230]
	in vivo Male Wistar rats treated with testosterone intramuscularly	6-weeks old	Testosterone supplementation compromised blood-spinal cord barrier integrity by decreasing protein expression of several tight junction proteins in the spinal cord	[230]

### Sex differences in cerebral blood flow might arise early in life

CBF is at the root of brain function providing oxygen and nutrients through modulation of capillary perfusion in response to neuronal activation [92]. In humans, CBF gradually increases during the neonatal period to reach a peak during youth, then it starts decreasing with age [93]. This reduction is felt particularly by the default mode and executive networks, which are brain circuits highly involved in cognitive control, mood regulation and behavioral modulation, among others [94]. Sex differences in CBF have been reported in adolescence, with young healthy women showing 11–15% higher CBF than healthy young men, an effect suggested to arise, at least in part, via gonadal hormones ([94, 95], see [87] for review).

Investigation of sex differences in fetal and neonatal CBF is lacking, making it challenging to pinpoint their origin. It has been reported that estrogen receptor concentrations rise shortly (4 to 6 days) in the neonatal rat forebrain prior to rise of nitric oxide synthase (NOS) [96]. This enzyme produces nitric oxide, a potent vasodilator which regulates vascular tone and CBF, activity within the brain [97]. In vitro evidence suggests that estrogen signaling can mediate upregulation of NOS [98], suggesting a potential role for estrogen signaling in regulating NOS-associated developmental pathways [96]. Still, we can only speculate about a potential link between estrogen and the regulation of CBF through NOS changes during early postnatal stages, a critical time point for BBB maturation and establishment of hemodynamic responses [49]. The functional relevance of this relationship should be thoroughly examined, as it could contribute to our understanding of sexually dimorphic patterns of brain development and potentially offer insights into CBF disturbances in neurodevelopmental disorders (see Sects. "Sex hormones, cerebral blood flow and angiogenesis: unraveling sex differences in autism spectrum disorder" and "Sex hormones, cerebral blood flow and angiogenesis: unraveling sex differences in attention deficit hyperactivity disorder (ADHD)").

## Sex hormones, angiogenesis and barriergenesis signaling: current knowledge and hypotheses on sex-specific mechanisms underlying cerebrovascular development

### VEGF signaling

VEGF is crucial for embryonic angiogenesis and is also identified as a neurotrophic factor capable of stimulating neurogenesis [99–102]. The VEGF protein family, often referred to as VEGF-A, comprises several isoforms, including VEGF-B, VEGF-C, VEGF-D, VEGF-E and placental growth factor (PlGF) ([101, 103], see [104, 105] for review). The influence of VEGFs is mostly driven by their interaction with specialized tyrosine receptor kinases, VEGF receptor (VEGFR)-1 and VEGFR-2, but VEGF can also interact with neuropilines (NRP) [101, 103, 106]. VEGF binding to VEGFR-1/-2 launches canonical signaling pathways involved in cell proliferation, migration, survival and vascular permeability, and is implicated in vasculogenesis and angiogenesis [99, 101].

Constitutive knockout of Vegfr2 (Kdr-/- mice) results in whole-body abolishment of blood vessel formation, which is lethal in homozygous embryos (~ E9), supporting the necessity of Vegf signaling during embryogenesis [107]. Ligand deficiency in Vegf-/- and Vegf ± mice also induces animals [108]. Postnatally, Vegf signaling is readily upregulated in response to insults in rodents (ischemia, inflammation, etc.) [109–111]. Interestingly, the choroid plexus retains high expression of Vegf, seemingly contributing to high endothelial fenestration and permeability of this brain area later in life [112].

### VEGF signaling and sex hormones: potential links to BBB development

In vitro and adult animal studies confirm that estrogens, progesterone, and androgens have direct angiogenic properties in the brain [113–116] (see Table 2). For example, transgenic female mice lacking either ERα or ERβ present significantly reduced cortical cerebral capillary density, concomitant with downregulation of Vegf signaling in adulthood [113], indicating that both ER subtypes are important for brain angiogenesis. Further, work conducted in adult songbirds revealed that testosterone is important for cerebral angiogenesis in both males and females songbirds [117–119]. To date, no attention has been paid to the developing brain.

**Table 2:** In vitro and adult rodent evidence of sex hormone-mediated effects on Vegf

Hormone	Sex & model	Age	Finding	Refs
Estrogen	in vivo Females OVX rats implanted with a 17β-estradiol slow-release pellet + exposed to a recombinant human VEGF patch on the cerebral cortex	12–14 weeks old	Exposure to VEGF significantly increased BBB permeability in OVX + vehicle-treated animals. This effect was rescued by 17β-estradiol	[232]
	ERɑ or ERβ knockout female mice	15 week old	Constitutive deletion of both ERɑ or ERβ decreased levels of Vegf, Kdr and Flt-1 in frontocortical tissues	[113]
	in vivo OVX female mice orally treated with resveratrol (estrogen agonist) + middle cerebral artery occlusion (MCAO)	10–11 weeks	Resveratrol pretreatment elevated tight junction protein levels and BBB integrity, resulting in reduced subsequent ischemic brain injury. MCAO-induced elevation of Vegf was attenuated by resveratrol, suggestive of neuroprotective effects mediated in part by ERs	[233]
	in vivo OVX female rats subjected to global cerebral ischemia (GCI) + Treatment with GPER-1 agonist (G1) upon reperfusion by intracerebroventricular injection	Adult (age unspecified)	GCI induced BBB breakdown in the CA1, which was rescued by G1 treatment and concomitant with decreased Vegfa protein levels	[234]
	in vitro Primary human endometrial epithelial cells transfected with human VEGF promoter-luciferase reporter + Exposed to 17β-estradiol	N/A	Treatment induced a 3.8-fold increase in luciferase activity, demonstrating a regulatory role of 17β-estradiol in VEGF gene transcription	[114]
Progesterone	in vitro Primary human endometrial stromal cells	N/A	Genome-wide high-throughput sequencing of human endometrial stromal cells revealed a binding site for progesterone receptor B (PGR-B) upstream of the VEGF transcription start site Several angiogenesis-related genes were reported downstream of PGR-B, including VEGF	[115]
Testosterone	in vitro Human endothelial progenitor cells (EPCs) isolated from peripheral blood of healthy adult men, exposed to dihydrotestosterone (DHT)	N/A	DHT, a bioactive metabolite of testosterone, promoted proliferation, adhesion and migration of EPCs as well as VEGF secretion	[235]
	in vitro Primary human umbilical endothelial cells (hUVECs) exposed to testosterone	N/A	Testosterone impacted hUVEC cell migration through AR-dependent mechanisms	[116]

VEGF signaling modulates functions in other cell types, such as oligodendrocyte progenitor cells (OPCs) migration [120]. Although not directly part of the NVU, OPCs migrate along the vascular scaffold of newly forming blood vessels during development [121] and support integrity of the mature BBB [122]. Sex hormones have been found to interact with OPCs, through long-lasting actions of androgens during early postnatal development in rodents. Indeed, higher oligodendrocyte density and thicker myelin sheaths were observed in the brain of male vs female mice. This difference emerges between P0 and P10 precisely when a late wave of OPCs arise and start differentiating driven by dihydrotestosterone (DHT), a biologically active androgen-related metabolite derived from testosterone with potent AR affinity [123].

As mentioned earlier, the impact of sex hormones in CNS angiogenesis, BBB formation and VEGF signaling during fetal and neonatal development remains largely unexplored. However, findings in vitro or in adult rodent models, using constitutive gene knockdown approaches summarized in Table 2, suggest that sex hormones can directly promote VEGF signaling and angiogenesis, in a time- and dose-dependent manner. As such, it would be interesting to investigate if similar mechanisms are at play in the developing vasculature, since early sexual differentiation coincides with barriergenesis in rodents (Fig. 1). The cells from the NVU might be sensitive to hormonal fluctuations in early development, which could further contribute to sex differences in vascular development and BBB formation including through Vegf-related signaling.

### WNT signaling

The canonical Wnt/β-catenin pathway is crucial for BBB maturation [35]. This pathway is extraordinarily complex, comprising 19 different Wnt ligands signaling through 10 members of the Frizzled (Fzd) family of G-protein coupled receptors, as well as through Low-density lipoprotein receptor-related proteins (LRP), in particular LRP5 and LRP6 [124]. During mouse embryogenesis, neuronal progenitor cells express Wnt7a and Wnt7b in the developing forebrain, ventral brain regions and intermediate spinal cord. Conversely, Wnt1, Wnt3, Wnt3a and Wnt4 are expressed in the dorsal spinal cord and hindbrain [35]. Wnt ligands bind to Fzd receptors on the vascular endothelium, leading to β-catenin stabilization, subsequent nuclear translocation and transcription of target genes involved in cell proliferation, adhesion, morphogenesis, and other developmental processes [125, 126]. While expressed in a variety of tissues, loss of Wnt/β-catenin signaling is only detrimental for BECs. Wnt7b knockout is embryonic lethal (between E11.5 and 12.5), causing severe brain hemorrhaging and abnormal vessel morphology [35]. Progressive downregulation of canonical Wnt signaling occurs during postnatal development; however, this pathway remains critical to promote barrier maturation, tight junction formation and maintenance of BBB integrity throughout life [124, 127, 128]. In the adult CNS, dysregulation of Wnt/β-catenin signaling has been implicated in several disorders with neurodevelopmental and post-neurodevelopmental origins, such as ASD, ADHD, and schizophrenia as well as Alzheimer’s disease and multiple sclerosis [128–133]. Thus, modulation of this pathway has attracted therapeutic interest for some years [134].

### Wnt signaling and sex hormones: potential mechanisms favoring BBB maturation

To our knowledge, no evidence of sex differences in the Wnt/β-catenin pathway regarding BBB development has been reported. However, sparse evidence of hormonal interactions with Wnt ligands allows us to propose hypotheses on sex-specific mechanisms underlying BBB development. For example, Wnt4 is expressed in the mesenchyme (an embryonic connective tissue precursor of many cell types) of both sexes and has been pinpoint as an essential factor for sex determination. In mice, Wnt4 promotes the development of females characteristics but suppresses male reproductive system [135]. On the contrary, Wnt4 is downregulated in male mice testes around E11.5, as gonads emerge [135]. Its critical importance in embryonic ovarian development is supported by evidence of masculinization in Wnt4-mutant female mice [135]. In turn, overexpression of Wnt4 in male mice leads to defective androgen synthesis and lower testicular levels of testosterone. Again, this period coincides with barriergenesis in rodents (~ E10, Fig. 1), suggesting a potential influence of early sex determination on BBB formation. To support this idea, Wnt4 has been linked to proper choroid plexus development of the mammalian brain [136], which is imperative for formation and integrity of the CNS [137]. However, it remains unclear if sex- and cell-specific levels of Wnt4 during gonadal development could affect BBB development and function.

Dikkopf (Dkk) proteins have been identified as Wnt pathway regulators. Dkk1, recognized as a potent inhibitor of Wnt signaling [138], binds to LRP5/6 receptors, thus competing with Wnt binding and providing modulation of its transcription [138]. In the developing neurovasculature, Dkk1 levels are tightly controlled to allow proper neovascularization [139]. At later stages of embryonic development and postnatally, Dkk1 is expressed in the aorta [140] and can be detected in differentiating human endothelial cells in vitro. Interestingly, a transcriptomic study of human placenta revealed sexually dimorphic gene expression and positive correlation between placental estrogen/testosterone ratio and LRP6 [141]. Further analysis identified an ERα response element at the LRP6 gene promoter, highlighting estrogen’s capacity to modulate LRP6 and to potentially interact with Wnt signaling during fetal development [141].

Overall, the findings presented in this section only provide a loose and conjectural connection between sex hormones-mediated modulation of Wnt/β-catenin signaling, and we could not find any studies specifically focusing on the brain or developing vasculature. However, some associations have been proposed in neurodevelopmental diseases like ASD and ADHD (see Sects. "Sex hormones, cerebral blood flow and angiogenesis: unraveling sex differences in autism spectrum disorder" and "Sex hormones, cerebral blood flow and angiogenesis: unraveling sex differences in attention deficit hyperactivity disorder (ADHD)"), highlighting the need to focus on mechanisms underlying sex differences in healthy cerebrovascular development.

## Sex hormones, cerebral blood flow and angiogenesis: unraveling sex differences in autism spectrum disorder

### Autism spectrum disorder: background and sex differences

ASD is a complex neurodevelopmental disorder, with a worldwide prevalence of ~ 1% [142]. According to the Diagnostic and Statistical Manual of Mental Disorders (DSM-5), ASD emerges early in life and is characterized by alterations in social interactions and communication across multiple contexts, differences in sensory processing as well as restricted and repetitive behaviors, interests, and activities [143]. Studies consistently report higher prevalence of ASD in men compared to women, at a ratio of about 4:1 [142, 144]. While this number remains debated [145, 146], several theories have emerged to explain sex differences in ASD diagnosis and prevalence, such as the Extreme Male Brain [147], Female Protective Effect [148, 149], Female Autism Phenotype ([150, 151], see [152] for review), and, more recently, Female camouflage [153]. Most of these theories are based on reports of sex-specific features; for example, men with ASD display more externalizing symptoms such as repetitive behaviors and interests, aggressiveness, and hyperactivity/shortness of attention span ([154–158], see [152] for review) while women display more seizure-like and internalizing behaviors, including depressive and anxiety disorders [154–157]. Women also tend to be diagnosed later than men, possibly due to differences in cognitive and language abilities which remain undetected by current standardized diagnostic tools [155]. Broad sex differences exist in psychopathological comorbidities in ASD, with about 70% of individuals presenting at least one psychiatric condition or behavior outcome ([156], see [152] for review).

**Fig. 2:**
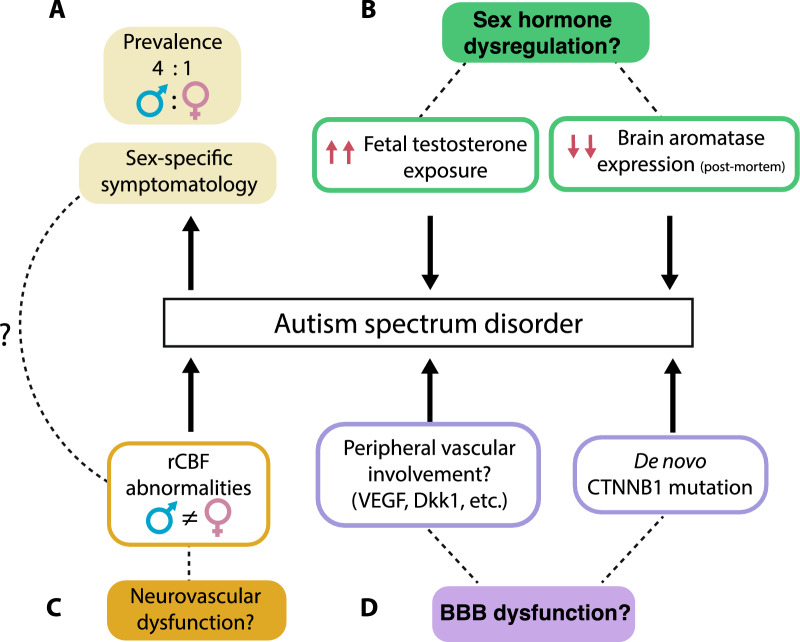
Sex hormones and cerebrovascular development may underlie sex differences in autism spectrum disorder in humans. **A** Autism spectrum disorder (ASD) is characterized by sex-specific prevalence and symptomatology. **B** Sex hormone dysregulation could contribute to it with reports of high prenatal testosterone exposure and decreased brain aromatase expression in individuals with ASD. **C** Patterns of regional cerebral blood flow (rCBF) in limbic brain regions are modulated by sex*diagnosis suggesting higher neurovascular coupling abnormalities in men with ASD, in line with the higher male-biased diagnosis ratio. **D** Sex differences in blood levels of VEGF and Dkk1 are present in individuals with ASD with higher peripheral VEGF levels in women. De novo mutations of CTNNB1 indicate a potential role for key vascular pathways (VEGF and Wnt/β-catenin) in ASD pathophysiology. Full arrows represent empirical findings and dotted lines highlight hypothetical associations. Sex-specific differences in cerebrovascular function and the dysregulation of key vascular pathways, such as VEGF and Wnt/β-catenin, may contribute to the sex-specific prevalence and symptomatology observed in ASD. This suggests that understanding the interplay between sex-related factors and cerebrovascular mechanisms could provide valuable insights into the underlying pathophysiology of ASD and its sex-specific variations

Several studies have shown that increased prenatal exposure to testosterone [159, 160], decreased aromatase expression, and reduced estrogen/ER expression (161–163) are correlated with ASD. For example, fetal testosterone levels have been associated with social development alterations in non-autistic toddlers and children; moreover, elevated fetal testosterone can predict poorer vocabulary size [164], decreased frequency of eye contacts [165] and lower empathy quotient scores [166] (Fig. 2). Similar findings were reported in children with ASD, with fetal testosterone being positively correlating with autistic traits [159, 167], thereby drawing a link between prenatal testosterone exposure and development of social characteristics (see [168] for review).

Furthermore, analysis of post-mortem brain tissue of individuals with ASD revealed significant reduction of CYP19A1 aromatase levels (Fig. 2) [162], an enzyme responsible for converting testosterone to estrogen in gonads and extra-gonadal sites (adipose tissue, bone, placenta, brain) [169]. This loss occurs in brain areas involved in executive functioning, attentional selection, and communication processes in social contexts [170–174]. It would be highly relevant to investigate whether decreased brain CYP19A1 levels (i.e. reduced aromatization of estradiol) leads to heightened CNS testosterone activity and if this contributes to the pathophysiology of ASD and associated sex differences.

### BBB and cerebrovascular dysfunction in ASD

The involvement of cerebrovascular deficits in the pathophysiology of ASD have been recently highlighted [175]. In humans, copy number microdeletions in the 16p11.2 locus were associated with ASD [175, 176]. Young adult male mice (P50) harboring constitutive 16p11.2 deletion exhibit reduced evoked CBF responses, concomitant with neurovascular uncoupling during stimulus-evoked whisker stimulation. In their elegant study, Ouellette et al. explored sex differences in the constitutive 16p11.2 haploinsufficiency model of ASD. Male mice with 16p11.2 haploinsufficiency only in BECs and VSMCs show impaired CBF, reduced angiogenic activity as well as delayed cortical vessel maturation which preceded neural abnormalities during postnatal development. Conversely, 16p11.2 mutant females mice displayed similar rates of angiogenesis between P14 and P50 when compared to sex-matched wild-type animals. Furthermore, behavioral phenotypes vary between sexes, with 16p11.2 haploinsufficient males showing sleep–wake cycle deficits and operant learning impairments when compared to females [175]. For the first time, this study showed fundamental differences in BEC biology that could contribute to concealing phenotypes in females; further, it raised the importance of investigating vascular dysfunction as a driving factor of ASD and highlights vascular-mediated mechanisms as contributors of sex differences in the pathophysiology of this disorder.

Cerebral perfusion deficits have also been reported in ASD and linked with behavioral patterns (Fig. 2A) [177–179]. Indeed, the interaction between sex and ASD diagnosis contributes to reduced regional CBF in limbic regions, including the subgenual anterior cingulate cortex, ventral striatum, and amygdala. Strikingly, changes in CBF in these areas were found to be greater in typically developing women than men, an effect reversed in ASD. These findings suggest that perfusion abnormalities are more common in men than women with ASD, in line with the skewed diagnosis ratio [180]. This is the only study we could find investigating sex differences and cerebrovascular function in ASD. Additional studies have correlated symptoms of impaired communication and social interaction with reduced regional CBF in the left medial prefrontal cortex and the anterior cingulate gyrus, but it has been observed in pooled ASD cohorts without sex stratification [178]. Overall, aberrant region-specific patterns of hypo- and hyperperfusion reported in ASD suggest a critical link between neurovascular coupling and ASD features, and affected brain regions may vary between sexes. It will be relevant to investigate the developmental origins of these differences and whether they underlie sexual dimorphism in ASD symptomatology.

### Possible hormone-mediated cerebrovascular influences in ASD

The above section highlights dysfunction of pathways involved in development, expansion, and maturation of the brain vasculature associated with ASD. No sex differences in brain VEGF levels have been reported so far (Fig. 2), but blood VEGF level was negatively correlated with symptom severity only in women [181], suggesting that peripheral angiogenic markers may be informative for ASD diagnosis and symptomatology. Currently, it is unclear if brain VEGF level and associated signaling may be impacted by those peripheral abnormalities. The potential involvement of VEGF in ASD-associated vascular dysfunction remains to be explored, as well as the effect of sex hormones on VEGF signaling in this context.

On the other hand, de novo mutations in the gene encoding β-catenin, CTNNB1, have been reported in ASD as well as in individuals with intellectual disability (Fig. 2), raising the idea of altered Wnt/β-catenin signaling in the pathophysiology of the disorder [182–184]. This concept is supported by rodent studies, with conditional Ctnnb1 gene knockout in parvalbumin neurons recapitulating core behavioral symptoms of ASD, including impaired social interactions and increased repetitive behaviors [183, 185]. Peripherally, serum levels of Dkk1, a potent Wnt pathway modulator, positively correlated with autistic trait severity in a young cohort of individuals (boys and girls) with ASD (Fig. 2) [186]. Platelets are a source of Dkk1, suggesting an immunomodulatory role for this protein in vascular inflammation [187]. Platelet activation and endothelium inflammation are observed in ASD [188], but potential sex differences are undetermined. Considering that peripheral inflammatory profiles are exacerbated in men vs women with ASD [189, 190], it would be relevant to investigate if alterations in serum Dkk1 could be stratified by sex and explore how peripheral levels of Wnt effectors could inform on brain endothelial and BBB dysfunction in ASD.

Altogether, cerebrovascular deficits are involved in the pathophysiology of ASD (Fig. 2), with promising research pointing to dysregulation of VEGF and Wnt pathways (Fig. 2). Influence of sex hormones, such as prenatal exposure to testosterone and decreased cortical aromatase expression are increasingly associated with the disorder and may contribute to its sexually dimorphic profile. Still, future studies are necessary to unravel the specific mechanisms underlying sex hormone-mediated contribution to vascular dysfunction in ASD. For example, could lack of estrogen-mediated immune protection in men result in increased platelet activation and Dkk1 release contributing to vascular inflammation in ASD? Or can spatial distribution of gonadal steroid receptors at the endothelium protect certain brain regions and underlie region-specific vascular dysfunction and sex-specific symptomatology in ASD? Better understanding of these mechanisms could inform future adapted treatment and development of better screening and diagnostic tools.

## Sex hormones, cerebral blood flow and angiogenesis: unraveling sex differences in attention deficit hyperactivity disorder (ADHD)

### ADHD: background and sex differences

ADHD is a psychiatric condition that emerges in childhood and preadolescence [191], affecting ~ 2–10% of children worldwide with a tendency to persist in adulthood in 2.5–7% of cases [192, 193]. The core symptoms of ADHD include hyperactivity, impulsivity, inattention, and distractibility [143]. These are thought to stem from abnormalities in executive functions such as decreased working memory performances or delayed reaction times, as well as disruptions of neuronal networks underlying cognition, attention, emotional processing, and sensorimotor functions [194]. Particularly, decreased activity and volume of the prefrontal cortex [195], structural and chemical abnormalities of the frontal lobe [195, 196], as well as CBF reductions have been described [197]. Despite the numerous studies identifying neurobiological and genetic factors associated with ADHD (see [198] for review), the precise etiology of the disorder remains debated. Diagnosis of ADHD is complex and often comorbid with other mood disorders, such as anxiety and depression, but also risk factors for cardiometabolic disorders like obesity, type-2 diabetes or dyslipidemia [199].

Sex differences are well established in ADHD [200], with higher prevalence observed in men vs women, at a ratio ranging between 4:1 and 9:1 for all ADHD spectrum disorders [201]. Interestingly, symptom severity is higher in women [201], and emerging research suggests that gonadal hormones underlie sex differences in ADHD symptomatology and prevalence [202–204]. In women, symptoms are exacerbated in the week preceding menstruation when levels of estrogen and progesterone decline [203]. Conversely, symptoms lessen during pregnancy when estrogen levels are heightened [202]. These findings align with previous research highlighting the positive influence of estrogen and progesterone on executive function and attention [205].

Indeed, high prenatal levels of testosterone have been proposed as a risk factor for ADHD. Studies have suggested that male and female rats exhibit different organization of dopaminergic circuitry, and dopaminergic innervation could be delayed in the striatum and prefrontal cortex of male rats [206]. This discrepancy has been linked to exacerbated testosterone levels during brain development [207]. Given the critical role of dopaminergic signaling in cognitive control [206], these provide intriguing mechanisms that may explain the higher prevalence of ADHD in men. Notably, most studies on brain development and ADHD do not test for sex differences, or simply lack power to conduct such analyses due to small sample sizes.

### BBB and cerebrovascular dysfunction in ADHD

As in ASD, further research is needed to elucidate the connection between brain vasculature and ADHD. An interesting approach involves spontaneously hypertensive rats (SHR), a widely studied animal model for cardiovascular diseases. SHR rats exhibit endothelial dysfunction as well as tight junction and caveolae modifications, albeit no significant increase in BBB permeability to albumin [194]. Interestingly, SHR rats display core symptoms of ADHD during development and adolescence, including inattention, hyperactivity, and impulsiveness [208–210], making it a suitable animal model to study vascular-related mechanisms in ADHD. It is worth noting that different strains of SHR rats display different phenotype profiles, allowing researchers to study different subtypes of ADHD.

A characteristic feature of ADHD is a reduction in CBF [211]. Although several neuroimaging studies have investigated regional volumetric and perfusion alterations in ADHD, exploration of sex differences remain sparse [87]. Volumetric alterations in men vs women with ADHD have been suggested [212], although these findings are not consistent with other work [213]. A current hypothesis proposes that alterations in CBF lead to energy deficits in the brain, subsequently altering neuronal functions and contributing to behavioral impairments associated with ADHD. The energy deficit hypothesis was explored by Medin et al. [214], who proposed that hyperactivity in children with ADHD is a compensatory mechanism for low lactate shuttling to the brain. Lactate, a byproduct of glycolysis, is a key metabolite involved in energy and brain homeostasis [214]. Using male SHR rats, Medin et al. [214] reported significantly higher levels of hippocampal lactate transporters in the SHR vs control animals, particularly at the BBB endothelium [214], suggesting an increased lactate shuttle from the periphery to the brain. This theory remains to be investigated in female rats. Interestingly, In women athletes, exhaustive physical exercise induces greater elevations of blood lactate levels and cortical excitability than men, potentially indicating a heightened sensitivity of women to cortical lactate levels [215]. Thus, it would be highly relevant to investigate sex differences in the energy deficit hypothesis of ADHD, and whether women exhibit fewer hyperactive behaviors than men due to better control of central energy levels through lactate shuttle in the BBB or sex different expressions of lactate transporters in the BBB. This mechanism could also be explored in the context of ASD.

### Possible hormone-mediated cerebrovascular influences in ADHD

To our knowledge, preclinical investigation of hormonal-mediated influences on angiogenesis and ADHD-related behaviors have only been conducted in male rodents. Using male juvenile stroke prone SHR rats (SHRSP), *Jesmin and al.* observed a decrease in VEGF levels and associated signaling in frontal cortical regions compared to healthy rats [197]. SHRSPs, characterized by impaired attentional capacities and intact androgenic signaling, showed significant downregulation in cortical VEGF signaling, ERα, CYP19A1, and capillary density compared to genetic control rats without ADHD-like behaviors. Ablation of androgenic signaling through castration counteracted these effects, rescuing VEGF levels and attentional capacities compared to intact SHRSPs [197]. Thus, androgenic hormone levels during specific periods of frontal cortex development may influence VEGF signaling and impact executive functions in SHRSPs juvenile males rats [197]. In humans, *Smith *et al. reported an interaction between single nucleotide polymorphisms (SNPs) in NRP1 and NRP2, two receptors for VEGF, and birth weight percentile in predicting the severity of hyperactive-impulsive symptoms [216]. Although this study included both men and women participants, sex differences were not investigated. While this early preclinical, and clinical work underscores a role for VEGF signaling in ADHD, future studies should focus on deciphering how sex as a biological variable influences these processes during critical periods of brain development.

Impairments in canonical Wnt/β-catenin signalization have also been linked to the pathophysiology of ADHD. Epidemiological reports point toward Wnt-related involvement in behavioral regulation, including hyperactivity [217–219]. A connection has been proposed between LRP5 and LRP6, two essential Wnt receptors, and cognitive impairment [129]. Specifically, a meta-analysis revealed significant association between *LRP5* rs3736228 gene variant with ADHD in girls, while *LRP6* rs2303685 gene variant was predominantly associated with ADHD in boys, suggesting sex-specific Wnt-related regulation underlying ADHD pathophysiology. Additionally, higher gene expression of both LRP5 and LRP6 is detected during brain developmental stages compared to adulthood [220], emphasizing the crucial role of this pathway in the brain particularly during development and maturation, which may contribute to its implication in ADHD [221]. As mentioned, a link has been established between placental estrogen/testosterone ratio and levels of LRP6 a gene carrying an ERα response element [220]. The functional effect of LRP5/LRP6 polymorphisms in ADHD and how estrogen may modulate these receptors remain unknown. Future work should elucidate whether sex hormones could be implicated in sexually dimorphic Wnt signaling underlying neurodevelopmental disorders such as ADHD, and how those effects tie in with cerebrovascular development.

**Fig. 3:**
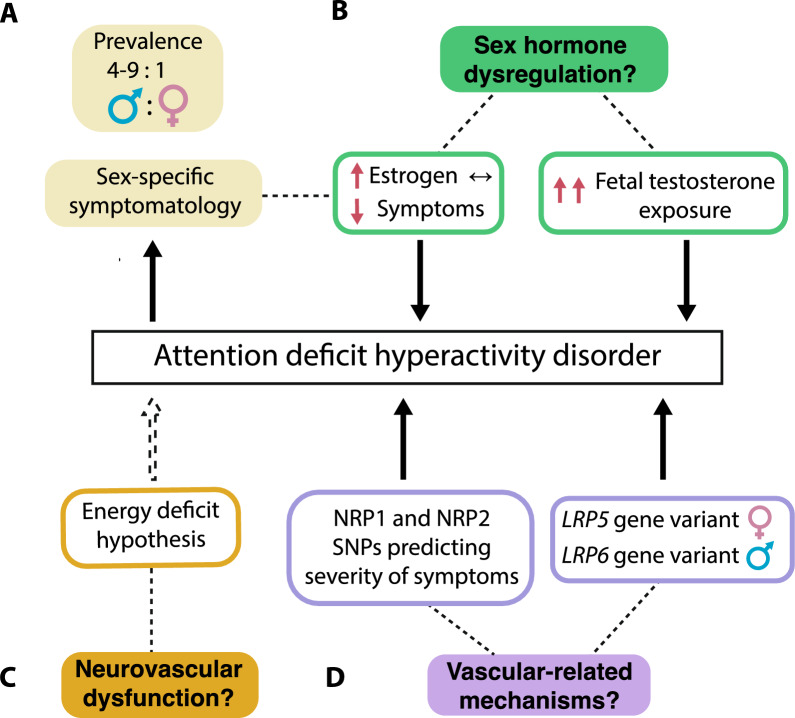
Sex-related differences in attention deficit hyperactivity disorder and potential influence on blood–brain barrier development. **A** Attention deficit hyperactivity disorder (ADHD) is more prevalent in men than women at a ratio ranging between 4:1 and 9:1, and sex differences are reported not only in symptomatology but also in symptom severity. **B** Sex hormones may be implicated in ADHD pathophysiology. Increased circulating estrogen (during pregnancy, for example) has been associated with dampening of symptoms. Moreover, high prenatal testosterone exposure is a risk factor for ADHD. **C** Motor hyperactivity could represent a compensatory mechanism for low lactate shuttling into the brain via the BBB. **D** Single nucleotide polymorphisms (SNPs) of two vascular endothelial growth factor (VEGF) receptors, NRP1 and NRP2 interact with birth weight percentile predicting severity of hyperactive-impulsive symptoms. Likewise, sex-specific gene variants of two essential Wnt receptors, LRP5 and LRP6 are reported in children with ADHD, possibly implicating BBB-related alterations in this disorder. Full arrows represent empirical findings and dotted lines represent hypothetical associations. Considering the data available, we postulate that cerebrovascular factors, influenced by sex differences, may contribute to variations in the development and severity of ADHD symptoms, potentially highlighting a novel avenue for understanding and treating ADHD

Altogether, the implication of sex hormones in the male-dominant prevalence of ADHD is increasingly accepted. Despite growing evidence of BBB and cerebrovascular dysfunction in this disorder, understanding of potential relationships is severely lacking. Still, promising findings such as sexual dimorphic Wnt-related gene variants, and implication of sex hormones in modulating regional CBF, underline the importance of including sex as a biological variable in ADHD cerebrovascular research (Fig. 3). Additional investigation is required to fully understand how sex hormone-driven mechanisms impact the development and maturation of the BBB and how these processes may be linked to sex-related differences in the clinical manifestation of ADHD. This includes exploring the influence of gonadal hormones on signaling pathways related to angiogenesis and cerebrovascular development, and their potential role in shaping sex-specific features of ADHD.

## Conclusion and future considerations

The contribution of brain vasculature in the pathophysiology of ASD [175, 177–179] and ADHD [194, 211] has only been recently described, paving the way for exploration of vascular specific mechanisms in neurodevelopmental disorders. With stark sex differences observed in symptoms, prevalence, severity, and treatment responses of both conditions [155, 158, 201, 202, 222, 223], it is imperative to dig further into sex-mediated processes in BEC development and BBB maturation. It should be highlighted that most of the preclinical studies in this area have been conducted in male animals, with findings often generalized to females despite growing knowledge of sex differences in physiological brain development, behavioral regulation, or even cerebrovascular functions in adulthood [224]. As we establish sex-specific profiles of neurodevelopmental disorders, developing models that replicate psychopathological traits for each sex is crucial. This will allow researchers to gain precise mechanistic insights on disorders mechanisms and to develop adapted treatments.

While cerebrovascular formation and barriergenesis occurs in parallel with sexual differentiation, minimal attention has been given to a possible relationship leading to sex differences in the maturation of brain vasculature and long-lasting distinctions [80] (Fig. 1). As mentioned, prenatal exposure to sex hormones has been associated with pathophysiological aspects of ASD and ADHD. However, hormonal influence on brain development and barriergenesis in health and disease can be complex. First, sex hormones are not only produced directly from the fetus following gonad formation but can also arise from the mother’s bloodstream by diffusion through the placental barrier (hormones or byproducts metabolized into hormones following entry in the placental or fetal compartments) [225, 226]. Consequently, shifts in the maternal–fetal environment during gestation likely influence prenatal cerebrovascular development (see [227] for review).

Second, it is important to reinforce that estrogens and androgens are not purely male and female hormones, respectively. Both sexes express ERs and ARs at their vasculature, and testosterone can be metabolized into ER agonists (see [83] for review). The functional implication of androgen signaling in females, and estrogen signaling in males, at the brain vasculature is poorly understood, let alone in a developmental context. Further complicating the matter is the ability for sex hormones to exert time- and dose-dependent effects on tight junction expression, angiogenesis, and vascular tone (see Tables 1 and 2). Expanding knowledge of sex- and brain region-specific distribution of gonadal hormone receptors at the BBB as well as their contribution from development through adulthood would undoubtedly contribute to elucidate the complex etiology of several diseases characterized by cerebrovascular dysfunctions.

This review has brought to light significant gaps in fundamental and clinically relevant knowledge regarding the possible involvement of sex hormones on BBB formation and cerebrovascular development. In virtue of the scarcity of available literature, we have proposed potential mechanisms for the development of the BBB and cerebrovascular function that could be influenced by sex hormones. These mechanisms include the VEGF and Wnt-β-catenin pathways, as well as regional CBF. We have integrated these concepts within the context of ASD and ADHD and have highlighted promising areas for future research. We offer several hypotheses that should not be construed as established facts but rather as subjects for further exploration in upcoming studies. Elucidating these mechanisms could hold the key to unlock novel screening tools and pharmacological therapies for neurodevelopmental disorders.

## Data Availability

Not applicable.

## Abbreviations

ADHD: Attention deficit hyperactivity disorder
AR: Androgen receptor
ASD: Autism spectrum disorder
BBB: Blood–brain barrier
BEC: Brain endothelial cell
CBF: Cerebral blood flow
CNS: Central nervous system
CTNNB1: Gene encoding β-catenin
DHT: Dihydrotestosterone
Dkk: Dikkopf
DSM: Diagnostic and statistical manual of mental disorders
E: Embryonic day
EC: Endothelial cell
EPC: Endothelial progenitor cell
Erα: Estrogen receptor alpha
Erβ: Estrogen receptor beta
Fzd: Frizzled
GCI: Global cerebral ischemia
GPER1: G-protein coupled receptor 1
HUVEC: Human umbilical endothelial cells
HPG: Hypothalamic-pituitary-gonadal
LRP: Low-density lipoprotein receptor-related protein
NOS: Nitric oxide synthase
NRP: Neuropilin
NVU: Neurovascular unit
OPCs: Oligodendrocyte progenitor cell
OVX: Ovariectomy
P: Postnatal day
PC: Pericyte
PDGF: Platelet-derived growth factor
PlGF: Placental growth factor
PNVP: Perineural vascular plexus
PGR: Progesterone receptor
SHH: Sonic hedgehog
SHR: Spontaneously hypertensive rats
SHRSP: Juvenile stroke prone SHR rats
SNP: Single nucleotide polymorphisms
VSMC: Vascular smooth muscle cells
VEGF: Vascular endothelial growth factor
VEGFR: Vascular endothelial growth factor receptor
